# An imaging study on establishing radial volar portals using the Kiss-in method in wrist arthroscopy

**DOI:** 10.3389/fsurg.2026.1835622

**Published:** 2026-07-01

**Authors:** Leining Wang, Jinhao Zhang, Bo Chen, Zhuolin Yuan, Bing Wang, Kunpeng Song, Gaoming Gong, Zhihui Huang, Rongzhang Qiu

**Affiliations:** 1 Department of Surgery of Hand and Foot, Beilun People's Hospital, Ningbo, China; 2 Department of Orthopedics, The First Affiliated Hospital, School of Medicine, Zhejiang University, Hangzhou, China; 3 Department of Nursing, Intensive Care Unit (ICU), Beilun People's Hospital, Ningbo, China

**Keywords:** imaging study, techniques utilization, volar portals, wrist anatomy, wrist arthroscopy

## Abstract

**Purpose:**

The feasibility and safety of the Kiss-in method for VRMC and VRRC portals in wrist arthroscopy were evaluated using 3D CT reconstructions in 64 adults (32M/32F) to quantify anatomical parameters and identify gender-specific risks.

**Methods:**

Mimics software generated 3D models of radius, ulna, carpal bones, radial artery (RA), median nerve (MN), and flexor carpi radialis (FCR). A 3.5 mm virtual trocar was inserted via the Kiss-in method; an axis-radius-aligned coordinate system enabled precise measurement of dTRA, dTMN, dTF, dTP, aTTR, aSTR, aNAT, and aNFT. A “risky zone” (≤0 mm from RA/MN) was defined and analyzed.

**Results:**

No statistically significant differences were found between most parameters of the two portals, except for dTP and dTF. The data for dTF, dTP, aTTR in the VRMC portal, and aSTR in the VRRC portal showed statistically significant differences between genders. Female dTP values at both joints were less than the trocar's length of 45 mm. Male dTP values in the VRRC portal exceeded 45 mm in 22 cases (68.75%), and in the VRMC portal, 10 cases (31.25%). The frequency of the “risky zone” was significantly higher in the VRRC portals.

**Conclusions:**

This imaging simulation study supports the theoretical feasibility of the Kiss-in method for establishing radial volar portals. The simulation results suggest potential safety benefits for the VRRC portal and indicate that the standard trocar length may be more adequate for female patients in this cohort. The determination of aTTR and aSTR has guiding significance for the practical application of the Kiss-in method.

## Introduction

**Figure 1:**
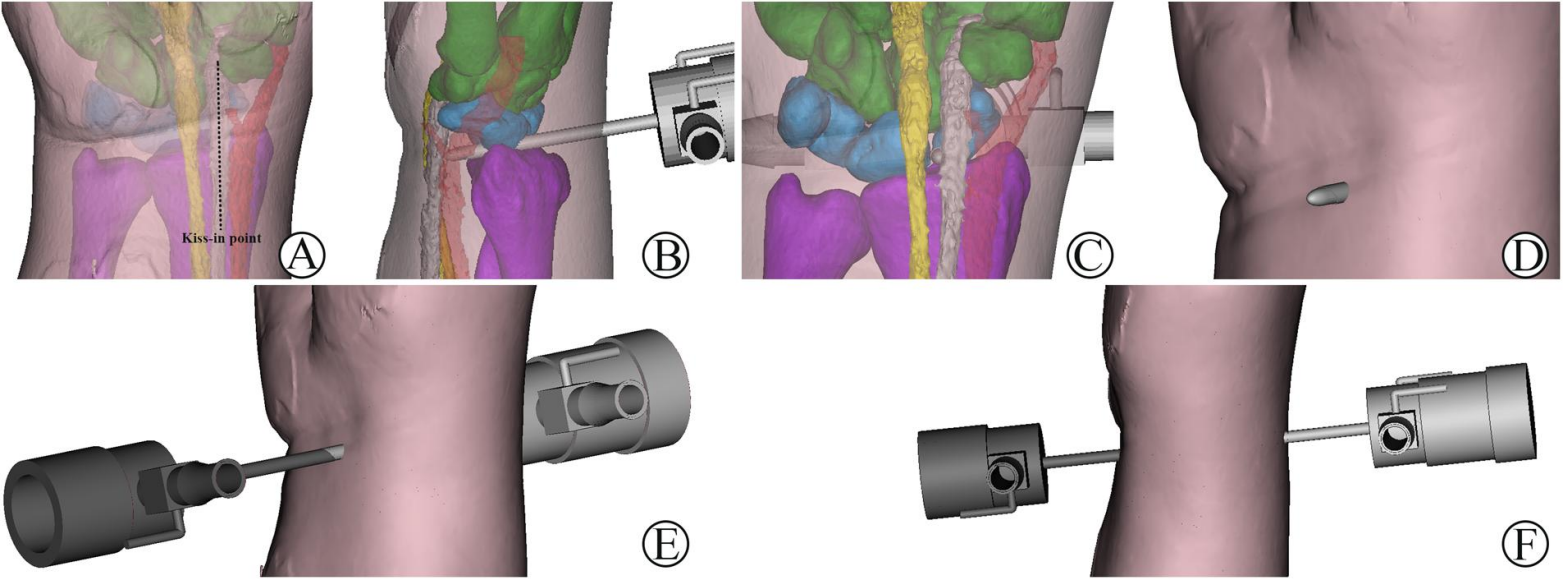
Establishment of the volar radial radiocarpal (VRRC) portal using the Kiss-in method in 3D reconstruction. **(A)** Surface palpation of the flexor carpi radialis to establish the axial line of the Kiss-in point; **(B)** Via the conventional 3/4 approach, the trocar tip penetrates the capsule reaching the center of flexor carpi radialis tendon; **(C)** Slightly slide the trocar tip toward the ulnar side, or displace the flexor carpi radialis tendon radially, to push the trocar through the space between the median nerve and FCR into the subcutaneous layer; **(D)** Advance the trocar out through the skin with a small incision; **(E)** Two trocar cannulas and one mandrel could fit each other and combine into a smooth pipe; **(F)** Push the trocar combination from the volar to dorsal into the radiocarpal joint to establish the VRRC portal.

Wrist arthroscopy has become a crucial technique for diagnosing and treating wrist disorders (1). Traditional palmar approach methods, such as the open operation (2), inside-out techniques (3, 4), each have distinct advantages and limitations. For instance, the open operation requires high surgical expertise and carries a risk of neurovascular injury with a 2-cm transverse incision. The inside-out technique involves complex procedural steps. The “Kiss-in” method (5), proposed by Dr. Chen Bo, is a novel palmar approach technique that has been preliminarily applied in wrist arthroscopy for ganglion cyst excision. Its core principle lies in precise pre-localization, allowing the trocar and sheath to “kiss” at a specific position, theoretically reducing the risk of injury to surrounding critical structures (e.g., radial artery, median nerve) and providing a smoother operative channel (Figure 1). The safety of this method lacks systematic imaging evaluation. The puncture trajectory in relation to the flexor carpi radialis (FCR), a crucial anatomical landmark, is a critical component of volar portal placement. The risk of neurovascular damage increases when a safe path is deviated from. However, there is no consensus on the optimal puncture path. The present imaging simulation study was designed to address this gap. It is crucial to clarify from the outset that the safety and feasibility data presented here are derived from an anatomical simulation model. Consequently, all resultant conclusions are theoretical and intended to lay an anatomical groundwork and assess technical plausibility before potential clinical application.

## Materials and methods

A retrospective collection of 64 adult wrist joint spiral CT scan data was conducted, including 32 male and 32 female participants aged 18–61 years. All participants underwent CT examination for mild wrist soft tissue injury or chronic wrist pain, without acute severe trauma, fracture, dislocation, ischemia, or degenerative arthritis. Cases with pathological anatomical changes, structural deformity, surgical history, or bony/carpal abnormalities were strictly excluded. All included wrists were from individuals with right-hand dominance. The cohort was distributed as: 17 left wrists and 15 right wrists in the male group, and 16 left wrists and 16 right wrists in the female group. All imaging data met modeling requirements, with soft tissue imaging clearly delineating the radial artery, median nerve, and flexor carpi radialis.

The original CT data of the 64 wrist joint scans were imported into Mimics 21.0 software (Materialise, Belgium). After reading the CT sequence images, thresholding techniques (*Threshold*) were applied using the standard bone tissue threshold (226–3,000 HU) to select the bony structures of the wrist joint, generating the initial mask. Subsequently, *3D mask editing* was performed to segment the excess midcarpal and radiocarpal joints. The *region-growing* tool was then used to obtain new masks for the radius and ulna, proximal carpal bones, and distal carpal bones. The radial artery, median nerve, and flexor carpi radialis were identified through manual segmentation, as the soft tissue thresholds were similar and non-uniform. After necessary cutting and smoothing of the masks, *3D calculation* was performed to generate the final models.

One set of CT data simulated the establishment of two volar radial portals for the midcarpal joint and radiocarpal joint (VRMC portal and VRRC portal) utilizing the Kiss-in method.

A standard 2.7 mm, 30° oblique sheath with a blunt mandrel was measured 45 mm length and 3.5 mm diameter.

**Figure 2:**
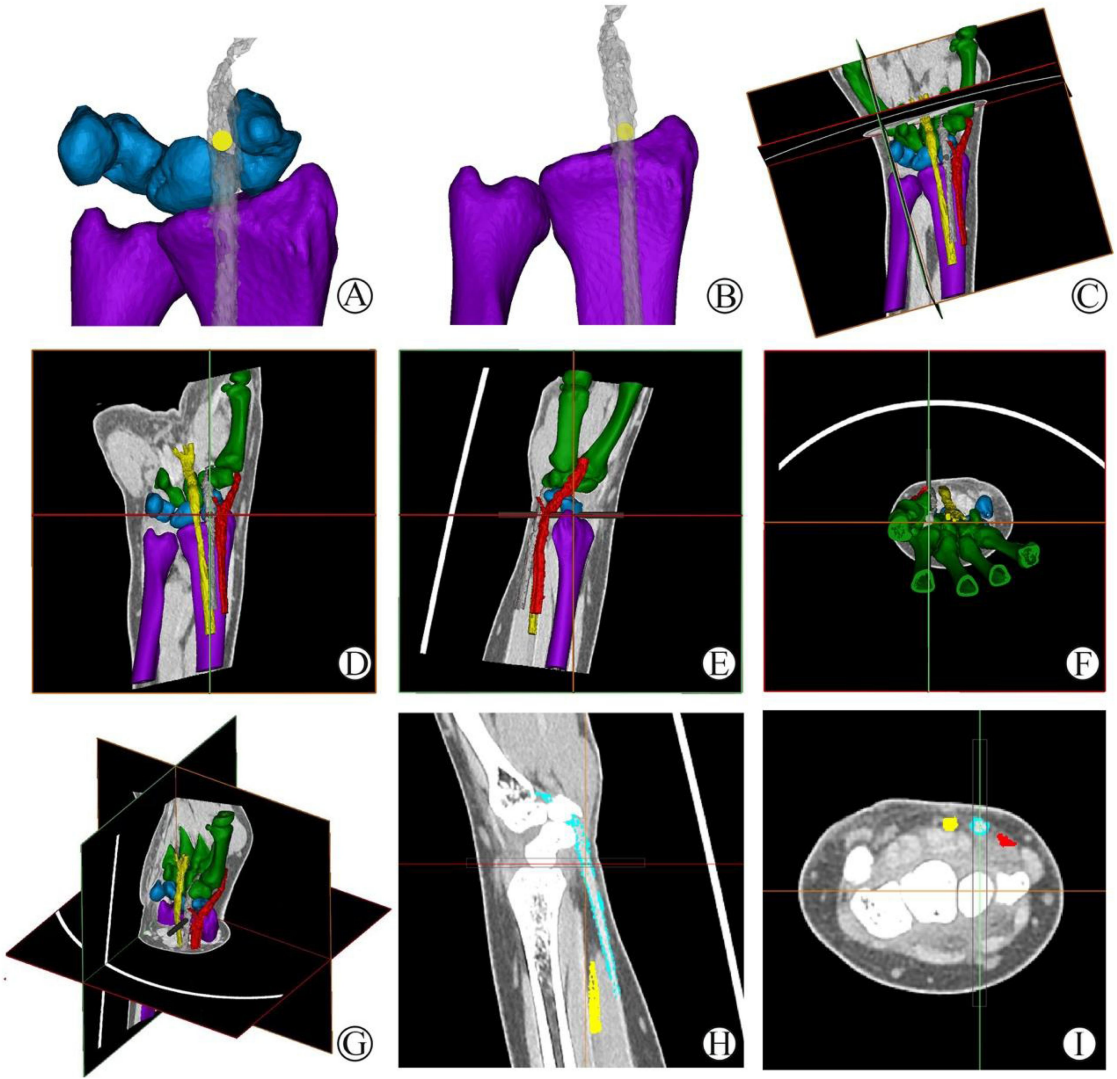
**(A,B)** The VRMC portal and the VRRC portal simulation, each cylinder placement was clearly followed that the trocar path avoids the joint bone surfaces; **(C)** Raw data coordinate system; **(D–F)** Coronal, sagittal, transverse planes in new coordinate system; **(G)** Isometric view of new coordinate; **(H,I)** Transverse and sagittal planes used for measurements in new coordinate system.

The VRMC portal: The distal row carpal bones were removed and a cylinder with a diameter of 3.5 mm, representing the wrist arthroscope trocar, was constructed. The dorsal entry point was located at the sagittal plane of the Lister tubercle, similar to the conventional dorsal radial midcarpal portal. The cylinder was placed on the proximal carpal bones, aligned with the center of the FCR tendon. If the bony structure was impassable, it was positioned as close as possible to the center of the FCR tendon (Figure 2A).

The VRRC portal: Both the distal and proximal row carpal bones were removed and a cylindrical structure with a diameter of 3.5 mm was constructed. The dorsal entry point was located at the sagittal plane of the Lister tubercle, similar to the conventional 3/4 approach. The cylinder was placed on the radius, aligned with the center of the FCR tendon (Figure 2B). Both portal cylinder placement was clearly followed that the trocar path avoids the joint bone surfaces.

**Figure 3:**
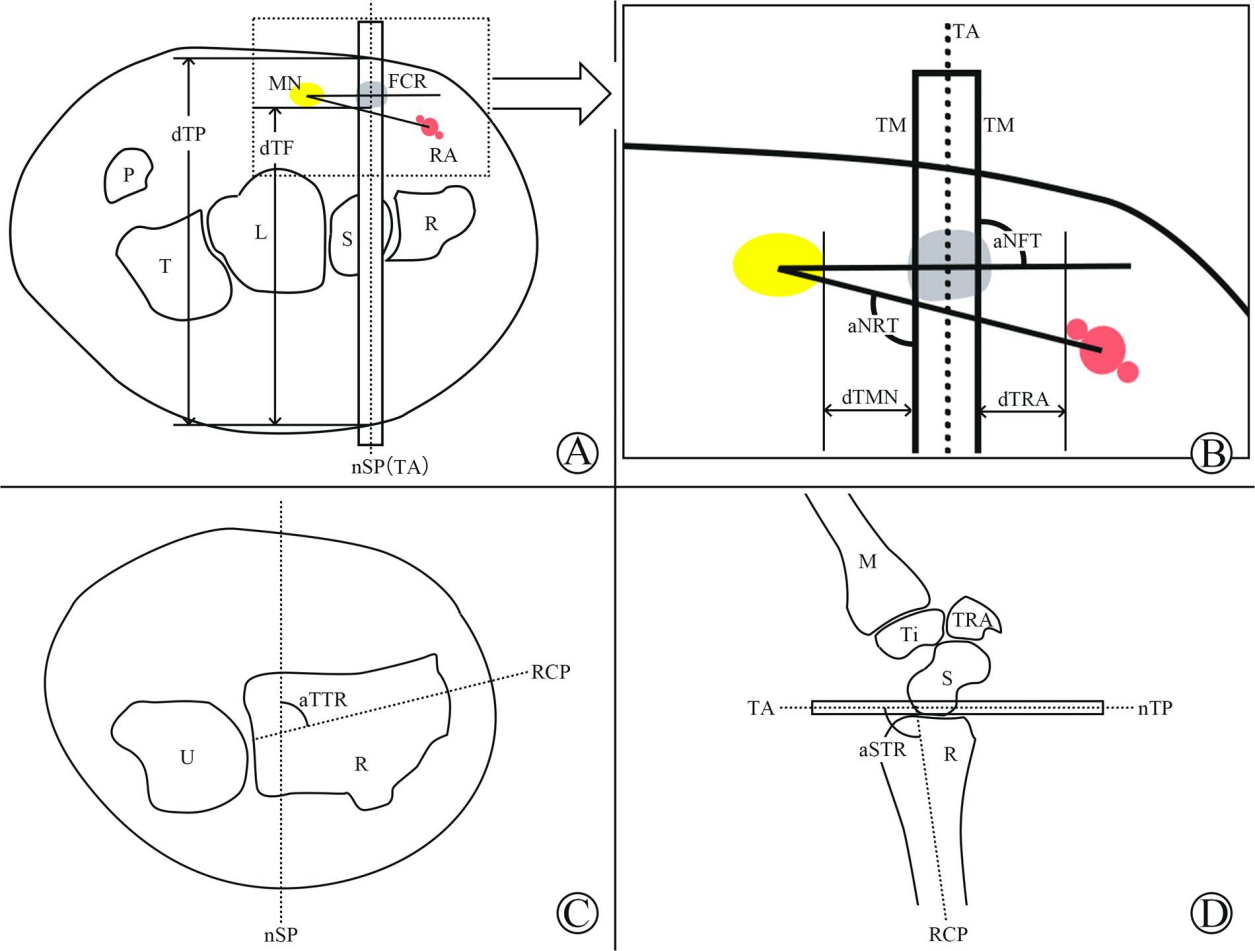
Illustration of measurement data. **(A)** Parameter measurements in transverse plane of new coordinate system; **(B)** Enlarged view of part **A**; **(C,D)** Measurements of the spatial angle between simulated puncture and the radial coronal plane; R, radius; U, ulna; S, scaphoid; L, lunate; T, triquetrum; P, pisiform; TRA, trapezium; Ti, trapezoid; M, second metacarpal bone; MN, the median nerve; RA, the radial artery or its nearby branch (superficial palmar branch), including accompanying veins; FCR, the flexor carpi radialis tendon; nSP, sagittal plane in new coordinate system; nTP, transverse plane in new coordinate system; TA, the trocar axis, paralleled to the nSP and nTP; TM, either trocar margin; RCP, coronal plane of the radius.

Using the *Reslice tool* to rotate and translate the coordinate axes, a new coordinate system passing through the cylinder axis and paralleled to the axis of the radius bone was reconstructed (Figures 2C–G). Relevant measurements were performed on the transverse and sagittal planes defined by this system (Figures 2H, I). The following data were measured and statistically analyzed (Table 1, Figure 3):

**Table 1:** Measurements in both portals.

Abbreviation	Explanation of the abbreviation
dTRA	Direct distance between the trocar margin and radial artery or its branch
dTMN	Direct distance between the trocar margin and median nerve
dTP	Axial depth of trocar insertion to palmar skin
dTF	Axial depth of trocar insertion to the center of the flexor carpi radialis
aNAT	Angle between the line from the median nerve center point to the radial artery or its branch center point and the trocar axis
aNFT	Angle between the line from the median nerve center point to the flexor carpi radialis center point and the trocar axis
aSTR	Sagittal angle between the trocar axis and the radius coronal plane
aTTR	Transverse angle between the trocar axis and the radius coronal plane
wRA	Width of the radial artery or its nearby branch (superficial palmar branch), including accompanying veins
wFCR	Width of the flexor carpi radialis tendon
wMN	Width of the median nerve

A “theoretical safety zone” was defined where both dTRA and dTMN ≥ 0 mm. Conversely, a “theoretical risky zone” was defined where either dTRA or dTMN < 0 mm. The distribution of the simulated trocars in these zones was recorded.

For both portals, the following spatial relationships were documented:

- Whether the FCR tendon was located dorsal to the MN-RA line.
- The spatial relationship between the MN-RA line and the coronal plane of the radius.
- For the VRMC portal: Whether the RA was immediately adjacent to the upper side of the FCR tendon.
- For the VRRC portal: Whether the radial artery had a branch in the vicinity.
- All simulation and measurement operations were performed by a single individual.

Statistical analysis was performed using paired student's *t*-tests to compare data (dTRA, dTMN, dTP, dTF, aNAT, aNFT, aSTR, aTTR) between the VRMC and VRRC groups. If a significant inter-group difference was found, independent student's *t*-tests were conducted to compare the same parameter between genders within that portal group. Chi-square tests were used to assess differences in categorical variables between genders and between portals. A *P*-value < 0.05 was considered statistically significant.

## Results

We successfully completed simulations and measurements for the midcarpal and radiocarpal joints across all 64 models. The results showed no significant age difference between male and female participants.

Paired data (dTRA, dTMN, dTP, dTF, aNAT, aNFT, aSTR, aTTR) for the different portals are recorded in Table 2. Inter-portal comparison revealed that the wRA) and the wFCR in the VRRC portal were significantly greater than those in the VRMC portal, while the wMN was smaller. Moreover, the spatial angle parameters (aSTR, aTTR) for the VRRC portals were significantly greater than those for the VRMC portals. The mean aSTR was 87.41° ± 1.42° and aTTR was 65.04° ± 0.89° in the VRMC group, compared to 102.65° ± 0.93° and 74.97° ± 1.12° in the VRRC group, respectively. The axial depth to palmar skin (dTP) was greater for the midcarpal joint (43.82 mm ± 0.70 mm) than for the radiocarpal joint (41.38 mm ± 0.71 mm). With the exception of dTF and dTRA, all other measured parameters demonstrated statistically significant differences between the VRMC and VRRC portals. Therefore, data from different portals were grouped separately for gender-based comparisons (see in Table 2).

**Table 2:** Comparison of measurements in different portals and genders.

Portals	VRMC			VRRC		
Gender	Male (*n* = 32)	Female (*n* = 32)	Sum (*n* = 64)	Male (*n* = 32)	Female (*n* = 32)	Sum (*n* = 64)
dTF (mm)	38.39 ± 0.59	33.94 ± 0.44*	36.16 ± 0.54	37.49 ± 0.80	33.26 ± 0.46*	35.38 ± 0.59
dTP (mm)	46.72 ± 0.86	40.92 ± 0.43*	43.82 ± 0.70	43.96 ± 0.95	38.80 ± 0.54*	41.38 ± 0.71*
wRA (mm)	3.51 ± 0.30	2.93 ± 0.36	3.22 ± 0.24	5.87 ± 0.39	5.52 ± 0.24	5.69 ± 0.23*
wMN (mm)	5.61 ± 0.11	5.24 ± 0.23	5.43 ± 0.13	5.02 ± 0.15	4.61 ± 0.18	4.81 ± 0.12*
wFCR (mm)	6.24 ± 0.23	5.69 ± 0.25	5.96 ± 0.18	5.11 ± 0.17	4.60 ± 0.09*	4.85 ± 0.11*
dTRA (mm)	0.80 ± 0.28	1.16 ± 0.25	1.03 ± 0.18	1.34 ± 0.25	1.91 ± 0.34	1.63 ± 0.21
dTMN (mm)	2.53 ± 0.35	2.28 ± 0.30	2.40 ± 0.23	3.40 ± 0.29	2.85 ± 0.26	3.13 ± 0.20*
aNAT (°)	69.88 ± 1.93	72.63 ± 2.85	71.25 ± 1.71	103.68 ± 2.45	107.99 ± 2.45	105.83 ± 1.75*
aNFT (°)	74.53 ± 1.47	75.76 ± 2.00	75.14 ± 1.23	89.39 ± 1.98	90.95 ± 3.70	90.17 ± 2.07*
aSTR (°)	84.18 ± 1.71	90.64 ± 2.00*	87.41 ± 1.42	101.09 ± 1.21	104.21 ± 1.34	102.65 ± 0.93*
aTTR (°)	63.83 ± 1.17	66.25 ± 1.30	65.04 ± 0.89	72.40 ± 0.99	77.54 ± 1.83*	74.97 ± 1.12*

*P < 0:05 in Female vs. Male or *P* < 0:05 in Sum vs. VRMC.

In both the VRMC and VRRC portals, the axial insertion depths to the FCR tendon (dTF) and to the palmar skin (dTP) were significantly greater in males than in females (all *P* < 0.05).

The wRA and wMN showed no significant gender differences in most comparisons. However, the wFCR at the radiocarpal joint was slightly wider in males (5.11 mm ± 0.17 mm) than in females (4.60 mm ± 0.09 mm, *P* < 0.05).

Additionally, gender differences were observed for the sagittal angle (aSTR) at the midcarpal joint (90.64° ± 2.00° in females vs. 84.18° ± 1.71° in males, *P* < 0.05) and for the transverse angle (aTTR) at the radiocarpal joint (77.54° ± 1.83° in females vs. 72.40° ± 0.99° in males, *P* < 0.05).

In the VRRC portal simulation, the radial artery was found to bifurcate, forming the superficial palmar branch, in 24 cases (37.50%). Among these, the superficial palmar branch was positioned closer to the simulated trocar in 20 cases (31.25%). In the remaining cases where the RA had no proximal branch in the field, we observed in 1 case the RA passing deep to (beneath) radial aspect of the FCR tendon. The average distance from the trocar margin to the median nerve (dTMN) was 3.13 mm ± 0.20 mm, which is considerably greater than the trocar's radius (1.5 mm). In 62 cases (96.88%), the FCR tendons were located dorsal to the MN-RA lines, indicating that the trocar tip, when inserted, had passed beyond the vertical projection of the median nerve relative to the trocar axis (see in Table 3).

**Table 3:** Comparison of spatial relationships in different portals and genders.

		VRMC			VRRC		
Spatial Relationships	Y/N	Male (*n* = 32)	Female (*n* = 32)	Sum (*n* = 64)	Male (*n* = 32)	Female (*n* = 32)	Sum (*n* = 64)
The branch of RA was immediately adjacent to the FCR tendon above	Y	24	22	46	N/A	N/A	N/A
	N	8	10	18	N/A	N/A	N/A
RA had a branch	Y	N/A	N/A	N/A	8	4	12
	N	N/A	N/A	N/A	24	28	52
The FCR tendon was located above the RA and MN line*	Y	10	10	20	30	32	62
	N	22	22	44	2	0	2
Radial deviated between the RA-MN line and the coronal plane of the radius*	Y	22	26	48	32	32	64
	N	10	6	16	0	0	0
Risky zone*	Y	6	4	10	2	0	2
	N	26	28	54	30	32	62
The median nerve related risky zone	Y	2	2	4	0	0	0
	N	30	30	60	32	32	64

*P < 0.05 in Sum, VRMC vs. VRRC.

In the VRMC portal simulation, the radial artery or its major branch was positioned dorsal to the FCR tendon in the majority of cases (78.13%). In 12 cases, the main radial artery trunk was closer to the trocar than its branches were. The MN-RA lines were mostly radially deviated from the coronal plane of the radius (75%), and the FCR tendons were predominantly located below the MN-RA lines (68.75%). There were no significant gender differences in these categorical spatial relationships. The average distance from the trocar margin to the median nerve (dTMN) in the VRMC portal was 2.40 mm ± 0.23 mm.

While gender differences in these categorical counts were not significant, the spatial relationships themselves differed significantly between the two portals: (1) the relationship between the FCR tendon and the MN-RA line, and (2) the relationship between the MN-RA line and the radial coronal plane.

The frequency of the simulated trocar entering the predefined “risky zone” was significantly higher for the VRMC portals (10 cases) than for the VRRC portals (2 cases) (*P* < 0.05). However, within the VRMC risky zone cases, only 1 were attributable to proximity to the MN; the majority were related to the RA.

All female dTP measurements at both portals were less than this 45 mm length. In contrast, male dTP measurements exceeded 45 mm in 22 cases (68.75%, 41.35 mm–53.53 mm) for the VRMC portal, and in 10 cases (31.25%, 37.04 mm–49.71 mm) for the VRRC portal. But all male dTF was less than 45 mm.

## Discussions

Although systematic research dedicated exclusively to volar arthroscopic portals of the wrist remains scarce in the literature, several authors have reported a preference for utilizing one or more volar approaches (6). The principal advantage of establishing volar portals is the enhanced visualization of key anatomical structures that may be partially obscured when using conventional dorsal portals. This improved visual access is particularly beneficial for surgical procedures such as ganglion cyst excision, the management of intercarpal ligament pathologies, and the repair of certain intra-articular fractures (7–9).

Following simulation of the trajectory required to penetrate the volar capsule using the Kiss-in method for the VRMC portal, we observed that in some cases, the tips of the simulated trocars could rarely reach the radial or middle side of the FCR tendon. This limitation appeared attributable to bony obstruction by the scaphoid. Consequently, we propose a refinement of the theoretical “Kiss-in point,” suggesting it should be defined as passing through the central axis of the FCR tendon, rather than being strictly confined to its radial border. It is noteworthy that in clinical practice, surgeons may employ ulnar deviation of the wrist to maneuver past the scaphoid and achieve the intended trajectory.

Our study, utilizing three-dimensional imaging simulation, provides anatomical evidence supporting the theoretical feasibility of establishing both VRMC and VRRC portals via the Kiss-in method and estimates its simulated safety profile based on key distance parameters (e.g., dTRA, dTMN). Furthermore, it provides an anatomical refinement by indicating that the optimal target area (the Kiss-in point) is located within the central region of the flexor carpi radialis tendon. In contrast to conventional approaches, the Kiss-in method aims to create an operative channel that approximates a perfect cylindrical geometry more closely. The entry path generated by alternative methods, such as the Inside-out technique, can occasionally result in an irregular conduit, analogously described as a “junction between a cylinder and a slanted tube”. In contrast, the Kiss-in method, through its principle of precise coaxial alignment upon “kissing,” is theorized to produce a channel with walls that are more continuous and smooth, which could potentially reduce the occurrence of secondary soft tissue damage during trocar insertion. Moreover, its advantages are more pronounced when compared to the inside-out technique.

Previous studies have employed various techniques. Tham et al. used an “inside-out” method to establish the VRMC portal by penetrating the skin on the ulnar side of the FCR tendon, a method that lacks a stable exit point and may increase the risk of nerve injury in clinical practice. Naroura et al. utilized an “inside-out” technique with a mandrel to establish four volar portals in a cadaveric study. Gillis et al. defined an approach where a blunt trocar, directed over the scapholunate articulation and aimed at the scaphoid tubercle, was used to establish volar midcarpal portals via the “inside-out” technique; notably, the “blunt” trocar was found to be sufficiently sharp to pierce tendons in cadavers. Their study which used a standard 2.7-mm arthroscope, found that the median nerve fascia was pierced in one specimen and that the median nerve averaged only 1.0 mm away from the trocar for the VRMC portal. Lauren reported that the NanoScope was positioned an average of 1.8 mm away from the median nerve in the VRMC portal, with no nerve penetration observed in cadavers (10). Our study found that using the Kiss-in method made distance between the trocar margin and the median nerve bigger, averaged 2.40 mm ± 0.23 mm in the VRMC portal and 3.13 mm ± 0.20 mm in the VRRC portal. With respect to the RA, in the VRRC portal, as the trocar tip reached the FCR tendon, the RA was found beneath radial aspect of the tendon in only 1 case, and moving toward the ulnar edge of the FCR tendon did not affect the RA. In contrast, in the VRMC portal, the RA was more commonly located superficial to the FCR tendon.

In contrast to the “inside-out” technique which lacks a clear external landmark, the Kiss-in method explicitly uses the FCR tendon as a surface marker. Additionally, while the “inside-out” mandrel puncture must be performed under direct arthroscopic vision (often requiring three separate skin incisions for multiple portals), the Kiss-in method streamlines the process. The trocar assembly, comprising a scope sheath and a blunt mandrel, can, with compression of the palmar soft tissue, successfully establish both VRMC and VRRC portals in nearly all cases. This method appears more suitable for females, who generally have thinner palmar soft tissues. However, these anatomical differences (dTF, dTP, VRMC-aSTR and VRRC-aTTR) between genders are merely morphological statistical findings, and their direct clinical guiding value and prognostic significance remain unclear. But we have successfully applied the Kiss-in method to establish the VRMC portal for ganglion cyst excision in clinical practice, requiring only two small incisions, which is advantageous for patients desiring an aesthetic hand appearance (5).

This study utilized Mimics software for 3D reconstruction. While widely used in medical imaging and excellent for processing CT/MRI data to generate high-precision models—with extensive applications in bony reconstruction for finite element analysis (11) and surgical planning in fields like thoracic and cardiac surgery (12). Its application to the wrist soft structure presents specific challenges. Mimics reconstruction is most effective in scenarios with clearly defined, high-contrast tissue boundaries. However, CT-based reconstruction of the densely interwoven soft tissues of the wrist is difficult. Although bone reconstruction is straightforward, segmenting nerves, blood vessels, and tendons requires meticulous manual identification across transverse, sagittal, and coronal planes. Within the carpal tunnel, minimal variation in tissue Hounsfield units and extensive branching of distal structures further complicate the process. Consequently, we could only reliably segment larger structures such as the median nerve, flexor carpi radialis tendon, radial artery, and the superficial palmar branch.

Using MRI for reconstruction, while offering superior soft-tissue contrast, poses significant clinical drawbacks due to much longer acquisition times. The feasibility of using CT for this purpose is largely driven by its high imaging efficiency and speed, representing a significant clinical advantage. For structures with sufficient contrast, CT data can be effectively segmented using thresholding and region-growing tools in 3D software, as demonstrated in studies analyzing scapholunate ligament kinematics (13). Advancements in deep learning now enable fully automatic bone segmentation from four-dimensional CT (4DCT) datasets, a crucial step toward the dynamic assessment of wrist ligament lesions and carpal instability, automating the analysis of complex dynamic imaging data (14). Despite its inherent limitation of lower soft-tissue contrast compared to MRI, these developments underscore the evolving practicality of CT for functional musculoskeletal evaluation.

Our measurements indicated that the distances from the simulated trocar margin to the radial artery and median nerve were shorter in the VRMC portal compared to the VRRC portal. This narrower safety margin correlates with the trocar entering a predefined “risky zone” more frequently in the VRMC portal, indicating a potentially greater risk and necessitating heightened caution during VRMC portal establishment. It is important to note that no anatomical variants, such as a duplicated FCR muscle, were identified in our cohort, though such variations were documented (15, 16). Furthermore, our CT-based methodology had inherent limitations in delineating finer neurovascular structures. Key branches, namely the superficial palmar branch of the radial artery and the palmar cutaneous branch of the median nerve, could not be reliably segmented due to the insufficient soft-tissue contrast resolution of standard clinical CT scans for visualizing such small-diameter structures. Consequently, our safety analysis pertains primarily to the main trunks of the RA and MN. For comprehensive preoperative mapping that includes these critical branches, imaging modalities with superior soft-tissue resolution, such as high-resolution MRI or high-frequency ultrasound, would be required.

Regarding spatial relationships, the FCR serves as a critical and consistent surgical landmark on the volar wrist. The RA consistently courses along its radial side, while the MN is located on its ulnar side. The FCR tendon is located radial to the MN in all individuals, with a mean FCR-to-MN distance of 7.87 mm reported in one ultrasonographic study (17). This FCR-to-MN distance is bigger than our observations. Our hypothesis is that the larger ultrasound measurement was due to the probe pressing on the palmar side during examination. So mild palmar pressing is beneficial for safely establishing the volar radial portal without injuring the nerves.

Additionally, there are inherent methodological constraints to acknowledge. Manual image segmentation and measurements performed by a single researcher may introduce observer bias, and the reproducibility of these data has not been verified. CT offers clear bony visualization but poor soft tissue contrast, which increases segmentation uncertainty. As a static anatomical model, it cannot simulate intraoperative dynamic changes such as soft tissue deformation, neurovascular displacement and positional anatomical variations. Accordingly, the present findings are merely theoretical and require clinical validation. Additionally, the standard trocar may be too short for some patients; in obese males with thick palmar soft tissues, the trocar tip may fail to reach the volar subcutaneous area.

Notably, the ≤0 mm threshold for defining the danger zone is a theoretical anatomical index and cannot reflect intraoperative dynamic changes. Soft tissue displacement during trocar insertion means that small distances on static images do not indicate elevated injury risk. This standard should be regarded only as an anatomical reference rather than a direct clinical safety indicator. The small single-center sample may restrict the generalizability of these anatomical findings. Further large-sample, multicenter studies are needed to verify gender-related anatomical differences. We have emphasized that the present results should be interpreted cautiously due to the limited sample volume.

In conclusion, this imaging anatomical study of the Kiss-in method, by quantifying the spatial relationship parameters between the simulated trocar and neurovascular structures, demonstrates the feasibility and safety of this approach. The use of CT reconstruction simulation has provided specific guidance angles for the volar radial approach to both the midcarpal (approximately 75° transverse, 102° sagittal) and radiocarpal (approximately 65° transverse, 87° sagittal) joints, offering valuable directional reference for clinical surgery. These angles can be utilized intraoperatively to correct trajectory errors. Notably, practitioners using the “inside-out” method can also refer to these angular data to adjust their approach and mitigate neurovascular injury. And the Kiss-in method, leveraging the distinct surface landmark of the FCR tendon and providing quantifiable angular guidance, represents a promising technique for establishing safer and more reproducible volar radial arthroscopic portals.

## Data Availability

The raw data supporting the conclusions of this article will be made available by the authors, without undue reservation.
